# Supplementary material for: Computational design of thermostabilizing point mutations for G protein-coupled receptors
Source: eLife. 2018 Jun 21;7:e34729. doi: 10.7554/eLife.34729 (PMC6013254; doi:10.7554/eLife.34729)
Supplement: Supplementary file 3. [file elife-34729-supp3.docx]

**Key Resources Table**

| **Reagent type (species) or resource** | **Designation** | **Source or reference** | **Identifiers** | **Additional information** |
| --- | --- | --- | --- | --- |
| gene (human) | 5-HT2C | GenScript | N/A |  |
| cell line (*Spodoptera frugiperda*) | Sf9 | A gift from Dr. Beili Wu (SIMM, CAS) | N/A |  |
| antibody | HA Epitope Tag Antibody, Alexa Fluor 488 conjugate (16B12) | Thermo Fisher Scientific | Cat#A-21287 RRID: AB_2535829 |  |
| recombinant DNA reagent | pFastbac1 | A gift from Dr. Raymond C. Stevens, University of Southern California |  |  |
| sequence-based reagent | Primers for site-direct mutagenesis | This paper | N/A | Attached |
| chemical compound, drug | ergotamine D-tartrate | Sigma | Cat#45510-1G-F |  |
| chemical compound, drug | ritanserin | Tocris | Cat#1955 |  |
| chemical compound, drug | doxepine | Tocris | Cat#508 |  |
| chemical compound, drug | clozapine | Tocris | Cat# 0444 |  |
| chemical compound, drug | SB228357 | Tocris | Cat#1375 |  |
| chemical compound, drug | mesulergine | Tocris | Cat#1644 |  |
| software, algorithm | Prism | GraphPad Software | N/A |  |
| software, algorithm | ICM-Pro | http://molsoft.com |  |  |
| software, algorithm | MAFFT | https://mafft.cbrc.jp/alignment/software/ |  |  |
| software, algorithm | GPCRdb | http://gpcrdb.org |  |  |
| software, algorithm | DbD | http://cptweb.cpt.wayne.edu/DbD2/ |  |  |
| software, algorithm | libsvm | https://www.csie.ntu.edu.tw/~cjlin/libsvm/ |  |  |
| sequence-based reagent | >I62V-F | cctctccatcgtaGTAattatcattatga |  |  |
| sequence-based reagent | >G69A-F | cattatgacaataGCTggaaacattctc |  |  |
| sequence-based reagent | >H85E-F | aaagaaacttGAAaacgctaccaact |  |  |
| sequence-based reagent | >G103A-F | ccgatatgttggtcGCTctgctcgtgat |  |  |
| sequence-based reagent | >Y125K-F | GcccctgcctagaAAGttgtgtcccgtct |  |  |
| sequence-based reagent | >Y125V-F | gcccctgcctagaGTAttgtgtcccgtct |  |  |
| sequence-based reagent | >R157T-F | GttatgtcgccattACCaacccaataga |  |  |
| sequence-based reagent | >R157Q-F | gttatgtcgccattCAGaacccaataga |  |  |
| sequence-based reagent | >T169K-F | tcaatagtagaAAGaaggctattatgaaaat |  |  |
| sequence-based reagent | >A171L-F | tagtagaaccaagCTCattatgaaaat |  |  |
| sequence-based reagent | >I172A-F | AgtagaaccaaggctGCTatgaaaatcgccatt |  |  |
| sequence-based reagent | >I172F-F | agtagaaccaaggctTTTatgaaaatcgccatt |  |  |
| sequence-based reagent | >G184A-F | ctattagcatcGCTgtttcagtgcccat |  |  |
| sequence-based reagent | >N203D-F | aaaagtcttcgtaGATaacacgacatgcgt |  |  |
| sequence-based reagent | >F220I-F | cttattggttccATCgttgcatttttcat |  |  |
| sequence-based reagent | >F224Y-F | gttgcatttTACatccccctcac |  |  |
| sequence-based reagent | >C235F-F | tatcacctacTTTctcacaatttac |  |  |
| sequence-based reagent | >L236R-F | acctactgtAGGacaatttacgtt |  |  |
| sequence-based reagent | >V240A-F | AcaatttacGCTctgagaaggca |  |  |
| sequence-based reagent | >V240S-F | acaatttacAGTctgagaaggca |  |  |
| sequence-based reagent | >G314A-F | gagcaaggttctcGCTatcgttttcttcg |  |  |
| sequence-based reagent | >L333V-F | attacgaacatcGTAtcagtcctttgcgaa |  |  |
| sequence-based reagent | >K348A-F | aagttgatggaaGCTctgttgaacgta |  |  |
| sequence-based reagent | >C360N-F | attggctacgtgAATtcaggaatcaatcc |  |  |
| sequence-based reagent | >G362L-F | GgctacgtgtgctcaCTCatcaatcc |  |  |
| sequence-based reagent | >G362A-F | ggctacgtgtgctcaGCTatcaatcc |  |  |
| sequence-based reagent | >L370D-F | actcgtctacacgGATttcaacaagatctat |  |  |
| sequence-based reagent | >K373E-F | acacgctgttcaacGAAatctatagacgcgcg |  |  |
| sequence-based reagent | >I374D-F | CgctgttcaacaagGATtatagacgcgcgtt |  |  |
| sequence-based reagent | >I374T-F | cgctgttcaacaagACCtatagacgcgcgtt |  |  |
| sequence-based reagent | >Y375F-F | ctgttcaacaagatcTTTagacgcgcgttctcc |  |  |
| sequence-based reagent | >N381R-F | CgcgcgttctccAGGtaccttaggtgc |  |  |
| sequence-based reagent | >T67C-F | GtaatcattatcattatgTGTataggtggaaacattct |  |  |
| sequence-based reagent | >G103C-F | gatatgttggtcTGTctgctcgtgatg |  |  |
| sequence-based reagent | >A87C-F | gaaacttcacaacTGTaccaactactt |  |  |
| sequence-based reagent | >A171C-F | tagaaccaagTGTattatgaaaatcg |  |  |
| sequence-based reagent | >A98C-F | GtcactcgcgattTGTgatatgttggtc |  |  |
| sequence-based reagent | >A140C-F | tgttgttctcgaccTGTtcaattatgcacc |  |  |
| sequence-based reagent | >S163C-F | CccaatagaacacTGTaggttcaatag |  |  |
| sequence-based reagent | >N166C-F | cacagtaggttcTGTagtagaaccaag |  |  |
| sequence-based reagent | >N342C-F | GaaaagtcttgtTGTcaaaagttgatgga |  |  |
| sequence-based reagent | >L345C-F | tgtaaccaaaagTGTatggaaaagct |  |  |
| sequence-based reagent | >T369C-F | ActcgtctacTGTctgttcaacaagat |  |  |
| sequence-based reagent | >N372C-F | CgtctacacgctgttcTGTaagatctatagac |  |  |
| sequence-based reagent | >Y375C-F | ctgttcaacaagatcTGTagacgcgcgttct |  |  |
| sequence-based reagent | >I62V-R | tcataatgataatTACtacgatggagagg |  |  |
| sequence-based reagent | >G69A-R | gagaatgtttccAGCtattgtcataatg |  |  |
| sequence-based reagent | >H85E-R | agttggtagcgttTTCaagtttcttt |  |  |
| sequence-based reagent | >G103A-R | atcacgagcagAGCgaccaacatatcgg |  |  |
| sequence-based reagent | >Y125K-R | agacgggacacaaCTTtctaggcaggggC |  |  |
| sequence-based reagent | >Y125V-R | agacgggacacaaTACtctaggcaggggc |  |  |
| sequence-based reagent | >R157T-R | tctattgggttGGTaatggcgacataaC |  |  |
| sequence-based reagent | >R157Q-R | tctattgggttCTGaatggcgacataac |  |  |
| sequence-based reagent | >T169K-R | attttcataatagccttCTTtctactattga |  |  |
| sequence-based reagent | >A171L-R | attttcataatGAGcttggttctacta |  |  |
| sequence-based reagent | >I172A-R | aatggcgattttcatAGCagccttggttctacT |  |  |
| sequence-based reagent | >I172F-R | aatggcgattttcatAAAagccttggttctact |  |  |
| sequence-based reagent | >G184A-R | atgggcactgaaacAGCgatgctaatag |  |  |
| sequence-based reagent | >N203D-R | acgcatgtcgtgttATCtacgaagactttt |  |  |
| sequence-based reagent | >F220I-R | atgaaaaatgcaacGATggaaccaataag |  |  |
| sequence-based reagent | >F224Y-R | gtgagggggatGTAaaatgcaac |  |  |
| sequence-based reagent | >C235F-R | gtaaattgtgagAAAgtaggtgata |  |  |
| sequence-based reagent | >L236R-R | aacgtaaattgtCCTacagtaggt |  |  |
| sequence-based reagent | >V240A-R | tgccttctcagAGCgtaaattgT |  |  |
| sequence-based reagent | >V240S-R | tgccttctcagACTgtaaattgt |  |  |
| sequence-based reagent | >G314A-R | cgaagaaaacgatAGCgagaaccttgctc |  |  |
| sequence-based reagent | >L333V-R | ttcgcaaaggactgaTACgatgttcgtaat |  |  |
| sequence-based reagent | >K348A-R | tacgttcaacagAGCttccatcaactt |  |  |
| sequence-based reagent | >C360N-R | ggattgattcctgaATTcacgtagccaat |  |  |
| sequence-based reagent | >G362L-R | ggattgatGAGtgagcacacgtagcC |  |  |
| sequence-based reagent | >G362A-R | ggattgatAGCtgagcacacgtagcc |  |  |
| sequence-based reagent | >L370D-R | atagatcttgttgaaATCcgtgtagacgagt |  |  |
| sequence-based reagent | >K373E-R | cgcgcgtctatagatTTCgttgaacagcgtgt |  |  |
| sequence-based reagent | >I374D-R | aacgcgcgtctataATCcttgttgaacagcG |  |  |
| sequence-based reagent | >I374T-R | aacgcgcgtctataGGTcttgttgaacagcg |  |  |
| sequence-based reagent | >Y375F-R | ggagaacgcgcgtctAAAgatcttgttgaacag |  |  |
| sequence-based reagent | >N381R-R | gcacctaaggtaCCTggagaacgcgcG |  |  |
| sequence-based reagent | >T67C-R | agaatgtttccacctatACAcataatgataatgattaC |  |  |
| sequence-based reagent | >G103C-R | catcacgagcagACAgaccaacatatc |  |  |
| sequence-based reagent | >A87C-R | aagtagttggtACAgttgtgaagtttc |  |  |
| sequence-based reagent | >A171C-R | cgattttcataatACActtggttcta |  |  |
| sequence-based reagent | >A98C-R | gaccaacatatcACAaatcgcgagtgaC |  |  |
| sequence-based reagent | >A140C-R | ggtgcataattgaACAggtcgagaacaaca |  |  |
| sequence-based reagent | >S163C-R | ctattgaacctACAgtgttctattggG |  |  |
| sequence-based reagent | >N166C-R | cttggttctactACAgaacctactgtg |  |  |
| sequence-based reagent | >N342C-R | tccatcaacttttgACAacaagacttttC |  |  |
| sequence-based reagent | >L345C-R | agcttttccatACActtttggttaca |  |  |
| sequence-based reagent | >T369C-R | atcttgttgaacagACAgtagacgagT |  |  |
| sequence-based reagent | >N372C-R | gtctatagatcttACAgaacagcgtgtagacG |  |  |
| sequence-based reagent | >Y375C-R | agaacgcgcgtctACAgatcttgttgaacag |  |  |
